# Acute kidney injury: from clinical to molecular diagnosis

**DOI:** 10.1186/s13054-016-1373-7

**Published:** 2016-07-07

**Authors:** Claudio Ronco

**Affiliations:** Department of Nephrology Dialysis and Transplantation, International Renal Research Institute of Vicenza (IRRIV), San Bortolo Hospital, Viale Rodolfi 37, Vicenza, 36100 Italy

## Abstract

The RIFLE classification was introduced in 2004 to describe the presence of acute kidney injury (AKI) and to define its clinical stage, based upon the serum creatinine level and urine output. The same criteria, although slightly modified, are used in the other scoring systems AKIN and KDIGO. Mortality and morbidity remain high in AKI, suggesting that current diagnostic methods are suboptimal, poorly accurate, and often timely inadequate in detecting the presence of early kidney injury. Conversely, a growing body of evidence indicates that new AKI biomarkers can be used to both rule out AKI and to assess high-risk conditions or the presence of subclinical forms. Neutrophil gelatinase-associated lipocalin or cell cycle arrest biomarkers seem to be sensitive and specific enough to be used in conjunction with existing markers of AKI for better classifying renal injury as well as dysfunction. Improvements in diagnosis, risk identification, stratification, prognosis, and therapeutic monitoring may improve prevention and protection from organ damage and help to identify patients at risk, allowing individualized therapy. In this view, we may say that AKI diagnosis has finally moved from clinical to molecular level with potential benefits for the patients because similar progress has been shown in other disciplines.

The incidence of acute kidney injury (AKI) is increasing especially in hospitalized patients and particularly in the ICU due to major surgery, iatrogenic interventions, and sepsis. In such conditions, age and comorbidities make the kidneys more susceptible to various exposures and insults [1]. Diagnostic criteria based on oliguria and serum creatinine (SCr) seem inadequate to describe the wide spectrum of mechanisms and conditions of AKI (Fig. 1). The RIFLE, AKIN, and KDIGO classifications have made some important advancements [2–4] but they still rely only on urine output and SCr, precluding the possibility of a timely and accurate AKI diagnosis, and neglecting subclinical forms of kidney dysfunction and damage.

**Fig. 1:**
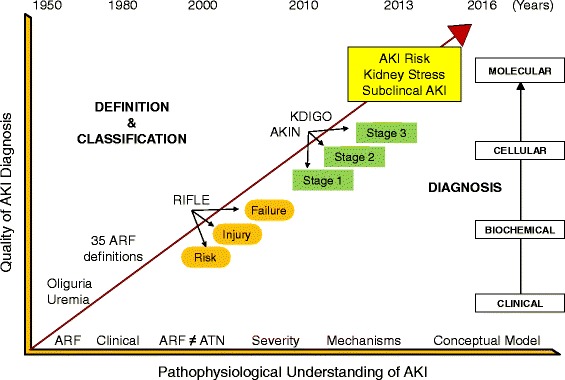
Evolution of AKI diagnostic syntax. The discovery and validation of cell cycle arrest biomarkers, neutrophil gelatinase-associated lipocalin, and other markers have permitted introduction of the concepts of AKI risk, kidney stress, and subclinical AKI. Quantitative evaluation of these markers has moved the diagnosis of AKI from the clinical/biochemical level to the cellular/molecular level. *AKI* acute kidney injury, *AKIN* Acute Kidney Injury Network, *KDIGO* Kidney Disease Global Outcome Initiative, *RIFLE* Risk, Injury, Failure, Loss and End Stage Kidney Disease, *ATN* Acute Tubular Necrosis, *ARF* Acute Renal Failure

New biomarkers can detect a risk of AKI or subclinical kidney damage earlier, allowing development of a new conceptual model for AKI with a continuum from initial kidney stress and early injury to advanced kidney damage and/or failure. The acute phase has also been called “Kidney Attack” [5] while the subsequent phases in the time window of 90 days are described as acute kidney disease (AKD). Full recovery or maladaptive repair with progression towards chronic kidney disease (CKD) is also a pathway described in the model. At each point of the continuum, biomarkers may play a role in clarifying mechanisms and clinical evolution of AKI. Studies on biomarkers have described their positive and negative predicting value for the presence and severity of the syndrome, site of damage, need for renal replacement therapy (RRT), and recovery or progression towards CKD [6]. Unfortunately, these studies present a high degree of heterogeneity, and meaningful conclusions are only obtained in specific populations.

Zhang et al. [7] conducted a meta-analysis focusing on the value of neutrophil gelatinase-associated lipocalin (NGAL) to predict AKI and clinical outcomes such as need for RRT and mortality in a specific subset of patients with sepsis. Fifteen studies were included in the analysis, confirming high-pooled sensitivity and specificity values for both plasma and urine NGAL. The authors concluded that NGAL is not only a good predictor of AKI but is also an efficient test to predict the need for RRT and mortality in septic patients.

Some questions remain open. Do we have discrete quantitative values correlated with level of damage/dysfunction or outcome? Can we diagnose AKI only in the basis of these molecular markers even in the absence of abnormal urine output or SCr? Does the cost/benefit ratio justify the use of these expensive biomarkers in critically ill patients? Are we ready to use these biomarkers routinely?

Not only can biomarkers be used to establish the presence and the severity of AKI, but they may also be used to identify a status of kidney stress or an increased susceptibility to insults. In such conditions, they may trigger early preventive and protective measures well before clinical AKI becomes manifest according to the KDIGO criteria [8].

The ADQI consensus group proposed the use of biomarkers to diagnose AKI with kidney damage even in the absence of renal dysfunction [9]. In a recent publication, de Geus et al. [10] concluded that NGAL is a good predictive marker for AKI in high-risk cardiac surgery patients and generated a NGAL score identifying a meaningful threshold for both plasma and urine NGAL values. The score contains memorable NGAL levels useful to rule out AKI or to quantify the degree of tubular damage. In the same line, the recent validation of discrete limits of cell-cycle arrest biomarkers (TIMP-2 and IGFBP-7) allows one to rule out AKI or to identify high-risk conditions for AKI. These biomarkers can describe a condition of kidney stress highly predictive of mild to severe AKI [11]. Biomarker levels may change over time, allowing identification of different phases of the syndrome such as increased susceptibility and risk, subclinical kidney damage, tissue regeneration, and recovery or progression towards CKD. Subclinical AKI may be diagnosed only with the use of biomarkers, when classic criteria are still within normal range (Fig. 2) [12, 13]. Specific biomarkers may represent a molecular signature for every type of insult (e.g., ischemia, sepsis, toxic elements, etc.). Moving from clinical to molecular diagnosis of AKI may allow characterizing the causative role of specific pathogenic factors and may help to develop individual criteria and decision-making frameworks for the etiological variants of AKI. AKI biomarkers are useful also to identify conditions of partial recovery, maladaptive repair, and progression towards CKD, important consequences of AKI also for health care providers. A cost/benefit ratio may be inferred by the application of biomarkers in specific populations. Implementation of routine use of biomarkers triggering specific alert conditions and rapid response strategies may help prevent evolution of AKI into more severe stages or requirement for RRT. If this happens even in a limited cohort of patients where RRT can be avoided or progression to CKD can be prevented, the financial advantage of biomarkers will become evident for patients and even for health care providers.

**Fig. 2:**
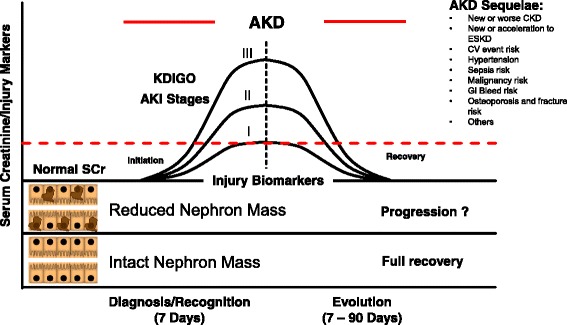
AKI is a short-term event that can, however, have sequelae up to 3 months (late recovery). A clinically manifest episode of AKI can be diagnosed by SCr or urine output allowing classification of patients into stages 1–3. Before that, however, a condition of initial or subclinical damage can be uncovered by injury biomarkers. The phase of recovery from AKI is somehow specular and while creatinine may come back to normal, partial or maladaptive repair can only be detected by injury biomarkers. The portion of the diagram below the line of normal SCr is potentially characterized by new biomarkers that allow a molecular diagnosis of AKI. *AKD* acute kidney disease, *AKI* acute kidney injury, *CKD* chronic kidney disease, *KDIGO* Kidney Disease Global Outcome Initiative, *SCr* serum creatinine

In conclusion, some of the key questions about biomarkers begin to find concrete answers [14]. The additional value of making an early and accurate diagnosis of AKI is gaining evidence and the role of biomarkers is increasing. Discrete quantitative values correlating with the level of damage/dysfunction are available and they will foster further studies of validation and support. Beyond the detection of increased risk and kidney stress conditions, today we can make a diagnosis of AKI based solely on molecular criteria even in the presence of normal urine output or SCr. The concept is mirroring what happened in the acute coronary syndrome in the absence of ST elevation on the EKG, where troponin made possible the diagnosis of non-ST elevation myocardial infarction. [15]. All of these considerations may suggest a remarkable cost/benefit advantage justifying the use of expensive biomarkers in specific populations. Thus, we will probably soon be ready to use AKI biomarkers routinely for the benefit of the patients and the strategic evolution of our healthcare plans.

## Abbreviations

ADQI, Acute Disease Quality Initiative; AKD, acute kidney disease; AKI, acute kidney injury; AKIN, Acute Kidney Injury Network; CKD, chronic kidney disease; EKG, electrocardiogram; KDIGO, Kidney Disease Global Outcome Initiative; NGAL, neutrophil gelatinase-associated lipocalin; RIFLE, Risk, Injury, Failure, Loss and End Stage Kidney Disease; RRT, renal replacement therapy; SCr, serum creatinine
